# Multiple tipping points and optimal repairing in interacting networks

**DOI:** 10.1038/ncomms10850

**Published:** 2016-03-01

**Authors:** Antonio Majdandzic, Lidia A. Braunstein, Chester Curme, Irena Vodenska, Sary Levy-Carciente, H. Eugene Stanley, Shlomo Havlin

**Affiliations:** 1 Center for Polymer Studies and Department of Physics, Boston University, 590 Commonwealth Avenue, Boston, Massachusetts 02215, USA; 2 Instituto de Investigaciones Físicas de Mar del Plata (IFIMAR), Physics Department, Universidad Nacional de Mar del Plata-CONICET, Funes 3350, 7600 Mar del Plata, Argentina; 3 Administrative Sciences Department, Metropolitan College, Boston University, Boston, Massachusetts 02215 USA; 4 Economics and Social Sciences Faculty, Central University of Venezuela, 1040 Caracas, Venezuela; 5 Department of Physics, Bar-Ilan University, 52900 Ramat-Gan, Israel

## Abstract

Systems composed of many interacting dynamical networks—such as the human body with its biological networks or the global economic network consisting of regional clusters—often exhibit complicated collective dynamics. Three fundamental processes that are typically present are failure, damage spread and recovery. Here we develop a model for such systems and find a very rich phase diagram that becomes increasingly more complex as the number of interacting networks increases. In the simplest example of two interacting networks we find two critical points, four triple points, ten allowed transitions and two ‘forbidden' transitions, as well as complex hysteresis loops. Remarkably, we find that triple points play the dominant role in constructing the optimal repairing strategy in damaged interacting systems. To test our model, we analyse an example of real interacting financial networks and find evidence of rapid dynamical transitions between well-defined states, in agreement with the predictions of our model.

## Abstract (web summary)

Systems composed of many interacting dynamic networks exhibit complicated collective dynamics. Here, the authors study failure, damage spread and recovery in two interacting networks, constructing the phase diagram and revealing the role of triple points for optimal damage repair.

## Graphical abstract

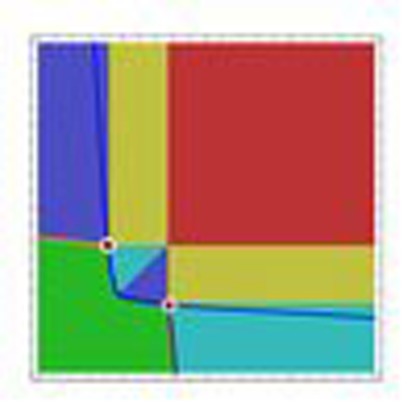

Most real networks are not isolated structures but interact with other network structures. As a result, much research has been focused recently on the dynamics of interdependent^1^,^2^,^3^,^4^,^5^,^6^,^7^,^8^ and multilayer^9^,^10^,^11^ networks. Recent studies on network repair^12^,^13^,^14^ have shown the importance of recovery of nodes as a process that leads to reverse transitions, hysteresis effects and such phenomena as spontaneous recovery^12^,^15^.

The cardiovascular and nervous systems in the human body are examples of two dynamically interacting physiological networks^16^. Diseases often result from complex pathological conditions that involve a dynamical interaction with positive or negative feedback between different functional subsystems in the body. Similarly, in the global economy there is a hierarchy of clustered and tightly connected countries, often grouped geographically, which are further interconnected to one large global interacting economic and financial network^17^,^18^,^19^. To understand the behaviour of these systems using network science, we develop a model of interacting networks with nodes that can recover from failure and we examine the resulting phase diagram.

Our model of a generic system consisting of interacting dynamical networks captures the important events found in real-world interacting networks, that is, node failure^20^,^21^,^22^,^23^, systemic damage propagation^24^ and node recovery^12^,^15^,^25^. We first analiticaly solve the model in the mean field theory (MFT) approximation and confirm the results in numerical simulations. The phase diagram of this system is characterized by a number of phases, phase transition lines and tipping points^26^,^27^,^28^,^29^. We show that triple points play a critical role in devising an optimal strategy for efficient repairing of interconnected network systems. By analysing the network of credit default swaps (CDSs) of sovereign countries, we demonstrate the application of our model to a real system and show that all of its parameters are experimentally accessible.

## Results

### Model

The structure of our system for the *n*=2 case is modelled as follows. We start with two isolated networks, network A and network B, and for simplicity we assume that both networks have the same number of nodes *N* and the same degree distribution *f*(*k*) (these assumptions can be relaxed but the results stay qualitatively similar). We assume that within each network the nodes are randomly connected. Now, to allow networks A and B to interact, we introduce interdependency links that connect nodes across the two networks^2^. This can be achieved in different ways and we use a simple one-to-one dependency: each node in network A is dependent on exactly one node in network B and vice versa. The pairs of nodes of both networks are chosen randomly.

The dynamic behaviour of our system is governed by two categories of event—failure and recovery—and we assume that every node is in either a failed or an active state. Node failure can result from internal failure or from the spread of damage from neighbour nodes in either the same network or the interdependent network. We thus assume that there are three ways a node can fail. The first way is the internally induced failure, when a node's internal integrity has been compromised, for example, an organ in the body can fail due to a malfunction within the organ or a company can fail due to bad management. The second type of failure is externally induced failure through failure propagation due to connections with failed nodes within the node's own network. Finally, there is a failure induced through the dependency link as a result of being dependent on a failed node from another (opposite) network. Apart of these three types of failures, we assume the existence of associated simple recovery processes for every type of failure. We specify quantitatively each of these processes below.

For internal failures (I), we assume that in both networks any node can fail due to internal problems, independent of other nodes. For each node in network A we assume that there is probability *p*_A_*dt* that the node will fail internally during any time period *dt*. The equivalent parameter in network B is *p*_B_.

Every node in network A and network B is connected by links to nearby nodes in its own network. These nodes constitute the node's neighbourhood. The number of links a node has within the network indicates its degree or connectivity, denoted by *k*. If a large number of nodes in a node's neighbourhood have failed, that is, if the neighbourhood is substantially damaged, we assume that the probability that the node itself will fail is increased. This is modelled by external failures (E). As in refs 12, 30, we use a threshold rule to define a substantially damaged neighbourhood, which is a neighbourhood containing ≤*m* active nodes, where *m* is a fixed integer threshold. If node *j* has >*m* active neighbours during time *dt*, we consider its neighbourhood to be ‘healthy' and there is no risk of external failure. On the other hand, if *j* has ≤*m* active neighbours during time *dt*, there is a probability *r*_A_ *dt* (for network A) or *r*_B_ *dt* (for network B) that node *j* will externally fail. For certain systems it is more appropriate to define a fractional threshold 0≤*m*_frac_ ≤1 as in ref. 15. That is, the minimum number of active nodes as a requirement for a ‘healthy' neighbourhood is replaced by a minimum fraction of active nodes in the neighbourhood. In the example of random regular network that we consider below, both are equivalent and related by *m*=*km*_frac_.

In the case of two interdependent networks (A and B), we assume that each node in the first network is dependent on a node in the second network via an interdependent link and vice versa. We assume that if one node in the pair fails, there is a finite (but not 100%) probability, *r*_d_ *dt*, that during time *dt* the other node in the pair will fail as well (dependency failure: type D). This represents the probability that the damage will spread through the interdependency link.

We also assume that there is a reversal process, a recovery from each of these three types of failure. A node recovers from an internal failure after a time period 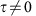, it recovers from an external failure after time 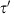 and from a dependency failure after time 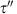. In simulations, and without loss of generality, we use 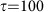 and for simplicity we set 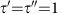, to take into account the assumption that real-world systems usually require a longer time period to recover from internal problems (physical faults) then from a lack of environmental support. However, changing the numerical values does not introduce any qualitative difference.

For the node activity notation, we assume that every node is in one of two states: active or failed. A node is considered active in the observed moment, if it is not experiencing internal (I), external (E) or dependency (D) failure.

### Mean field theory

We characterize this system by studying the order parameters chosen naturally as the fraction of active nodes in network A and network B, *z*_A_ and *z*_B_, respectively. To simplify the calculation, however, we first concentrate on the complementary and equally intuitive fraction of failed nodes *a*_A_ and *a*_B_, in networks A and B, respectively (*a*_A_=1−*z*_A_, *a*_B_=1−*z*_B_).

Using the MFT presented in Methods, we obtain two coupled equations that connect *a*_A_ and *a*_B_, which the system must satisfy in the equilibrium


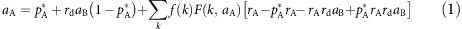



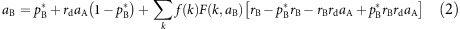


Here 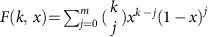 and we have also introduced simplifying parameters 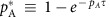 and 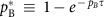, to make the equations more elegant and to reduce the number of parameters by replacing *p*_A_, *p*_B_ and 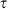 that appear as a product. We find that the parameters 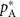 and 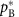 are very convenient to work with, because they correspond to the fraction of internally failed nodes in network A and network B, respectively.

Despite the seeming complexity of equations (1) and (2), it is noteworthy that there are only two unknown variables, *a*_A_ and *a*_B_, and that all other parameters are fixed. These two equations define two curves in the (*a*_A_, *a*_B_) plane.

**Figure 1:**
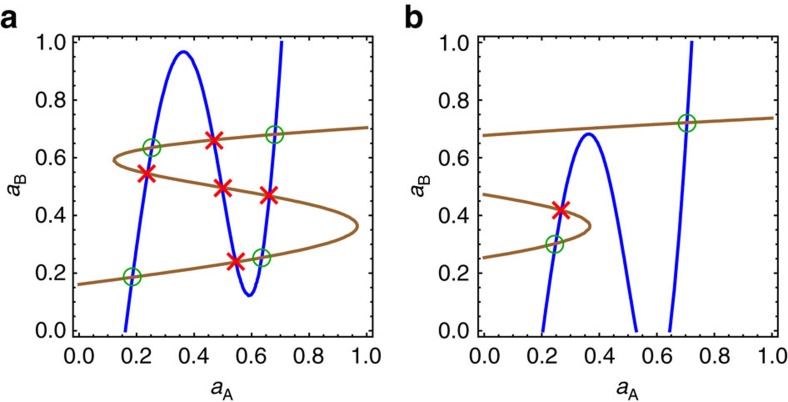
Graphical representations of the mean field equations. (**a**) The blue and brown curves represent equations (1) and (2), respectively, for 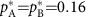, *r*_A_=*r*_B_=0.60 and *r*_d_=0.15, in a system with two interdependent networks (*k*=16, *m*=8). There are nine intersections, representing mathematical solutions for network activities *a*_A_ and *a*_B_. Four of them are stable solutions (green circles) representing physical states that we also observe in our simulations and five are unstable solutions (red crosses). (**b**) Example for 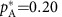, 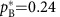, *r*_A_=*r*_B_=0.60 and *r*_d_=0.15. Here we obtain two stable solutions and one unstable solution. The two stable solutions correspond to 11 state (both networks are at high activity) and 22 state (both networks are at low activity).

Figure 1a shows a graphical representation of the curves for a random regular^31^ network (in which all the nodes have the same degree) with degree of *k*=16 and threshold *m*=8, for the symmetric parameter values 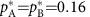, *r*_A_=*r*_B_=0.60 and *r*_d_=0.15. The size of each network is *N*=2 × 10^4^. The blue curve is a graphical representation of equation (1) and the brown curve is defined by equation (2). The curves, similar to two ‘ropes', create a ‘knot' that can have up to nine intersections, representing mathematical solutions of the system of equations. However, not all of these solutions represent observable and stable physical states. To see that, observe one of the curves in Fig. 1a, for example, the blue curve described by equation (1). If we increase damage done to network B (that is, we increase *a*_B_) and keep everything else constant, some damage will undoubtedly spread to network A. Thus, we expect that when *a*_B_ is increased, *a*_A_ must also increase (it would be very unusual if one network improves its activity as a result of damaging the other network). We conclude that the parts of the blue and brown curve that produce physical solutions are only those where *a*_A_ and *a*_B_ increase together or decrease together along the curve. This elimination leaves only four states in Fig. 1a that are stable (green circles), whereas the other five states are unstable (red crosses), for this particular choice of parameters. In simulated finite networks, when the network system evolves according to the rules of the model, at *t*=0 we have a freedom to set initial conditions for the activities. Systems initially prepared to have a pair of values (*a*_A_, *a*_B_) corresponding to an unstable solution of equations (1) and (2) will be disturbed by a small fluctuation of *a*_A_ or *a*_B_, owing to the system dynamics, and the values of *a*_A_ or *a*_B_ will rapidly change until one of the stable states is reached. Systems that are initially prepared to have values of *a*_A_ or *a*_B_ corresponding to a stable solution will fluctuate around these values, until perhaps a large finite fluctuation occurs and the system ‘jumps' to another stable state. In general, for any choice of parameters, we have between one and four stable (physical) states. Figure 1b shows the scenario for the same network system when 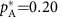, 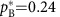, *r*_A_=*r*_B_=0.60 and *r*_d_=0.15. In this case we have two stable states and one unstable state.

In the Methods, section ‘Additional phase diagrams', we show diagrams for *z*_A_=1−*a*_A_ for a range of different values of 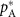 and all other parameters fixed. This MFT calculation agrees well with the states that we observe in our simulations, as we will demonstrate below.

The four stable solutions found above correspond to the following four scenarios: ‘11' or ‘up–up', when there is high activity in both network A and network B; ‘12' or ‘up–down' when there is high activity in network A and low activity in network B; ‘21' or ‘down–up' when there is low activity in network A and high activity in network B; and ‘22' or ‘down–down', when there is low activity in both network A and network B.

**Figure 2:**
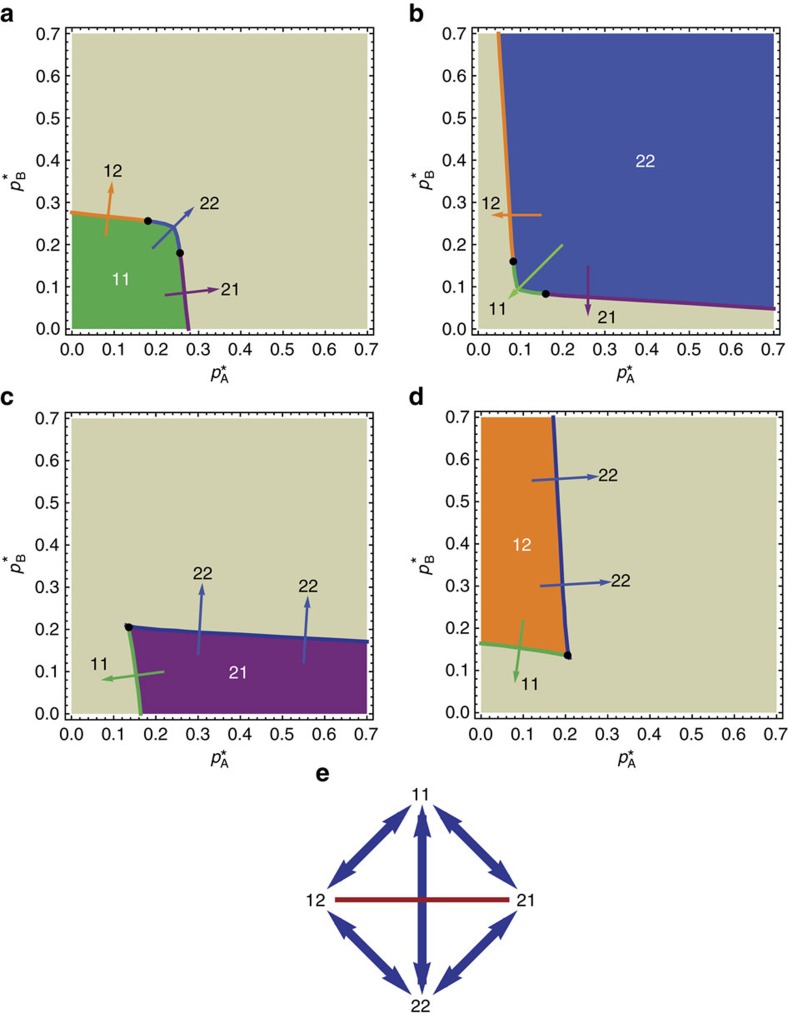
Four layers of the phase diagram and the transitions connecting them. (**a**) Region of 11 state, in green. Possible transitions are 11→12 (orange line), 11→22 (blue line) and 11→21 (purple line). This layer of the phase diagram has two triple points, marked as black points. (**b**) Region of 22 state (blue), with two triple points and three transitions. (**c**) Region of 21 state (purple), with two transition lines (to 11 and 22 state) that merge in a critical point. (**d**) Region of 12 state (orange), with two transition lines (to 11 and 22 state) that merge in a critical point. (**e**) Illustration showing states (11, 12, 21 and 22) with allowed (blue arrows) and forbidden (red line) transitions.

Depending on the parameters, we obtain between one and four stable states. Each of the states exists in a certain volume of the multi-dimensional space of parameters. Results of the MFT calculation for a particular set of parameters are presented in Fig. 2a–d as a phase diagram with four layers. Figure 2 shows the regions in which each of the four states exist in the (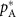, 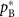) parametric sub-space, when other parameters are fixed at values *r*_A_=*r*_B_=0.60 and *r*_d_=0.15, with *k* and *m* remaining the same as before.

For example, in Fig. 2a the green area indicates the region where the 11 state exists. This state (phase) is bounded with a smooth boundary of three colours. If the boundary is crossed (by increasing 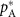 and 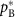), the system makes a transition to state 12 (if the orange line is crossed), state 22 (if the blue line is crossed) or state 21 (if the purple line is crossed). The arrows indicate transitions. In Fig. 2a, there are two triple points (black points) that mark the change in the transition type and where three different states can exist. The blue area in Fig. 2b indicates the 22 state. This layer of the phase diagram has two triple points as well and three possible transitions (22→12, 22→11 and 22→21).

Figure 2c,d show the regions of state 21 (purple) and state 12 (orange), respectively. Each has two different transitions and one critical point. For example, there are two possible ways out of state 21 (Fig. 2c): by a transition to the 11 (green arrow) state or the 22 (blue arrow) state. It is noteworthy that the different state regions (Fig. 2a–d) are not disjoint sets but there is an overlap, resulting in twofold, threefold or even fourfold hysteresis regions.

**Figure 3:**
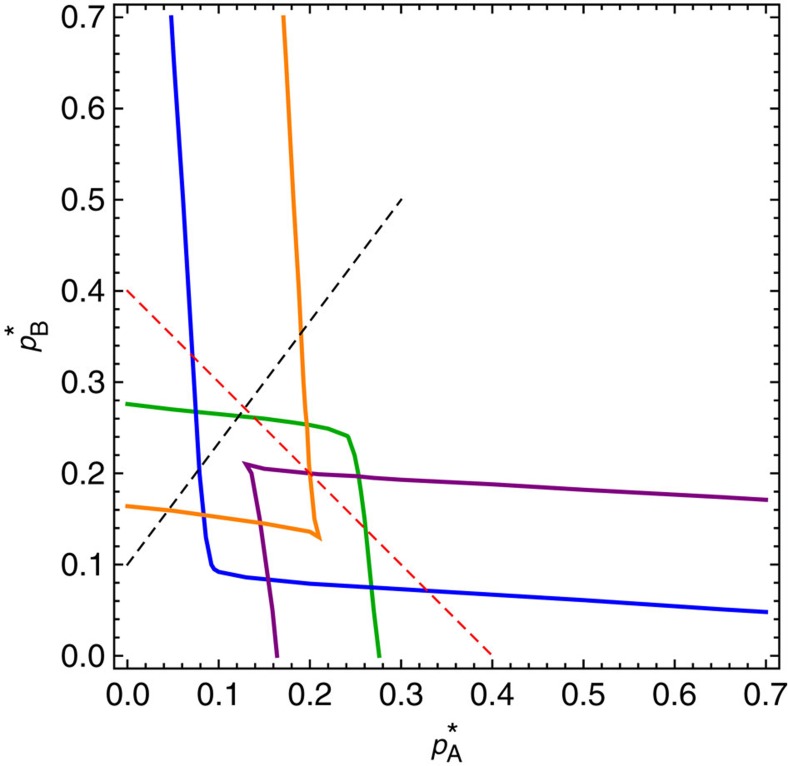
Total phase diagram with all four layers. Solid lines represent the borders of region 11 (green), 22 (blue), 12 (orange) and 21 (purple). Dashed lines represent cross-sections where we calculate the activity profile, shown in Fig. 4. It is noteworthy that there is a small central ‘window' where all four states are possible.

The state in which the system is found depends on the initial conditions or the system's past. There are a total of 10 different transitions (11→12, 11→22, 11→21, 12→11, 12→22, 21→11, 21→22, 22→12, 22→21 and 22→11) that connect different layers of the phase diagram (states 11, 12, 21 and 22), much similar to elevators connecting different floors. Transitions 12→21 and 21→12 are the only missing (‘forbidden') combinations. Although regions 12 and 21 do overlap, there is no direct transition connecting these two states. The set of all allowed and forbidden transitions is presented in Fig. 2e. The total phase diagram (all four layers on top of each other) is presented in Fig. 3. Here, coloured lines represent the boundaries of four states, with each colour corresponding to the boundary of one state, for example, the green line is a boundary of the 11 state. Rich critical phenomena with discontinuous hybrid phase transitions and second-order transitions have been recently discovered in multiplex networks. In particular, Baxter *et al.*^32^ introduced weak bootstrap percolation and weak pruning percolation in multiplex networks, which have potential applications in infrastructure recovery and information security, and can even provide a way to diagnose missing layers in a multiplex network.

**Figure 4:**
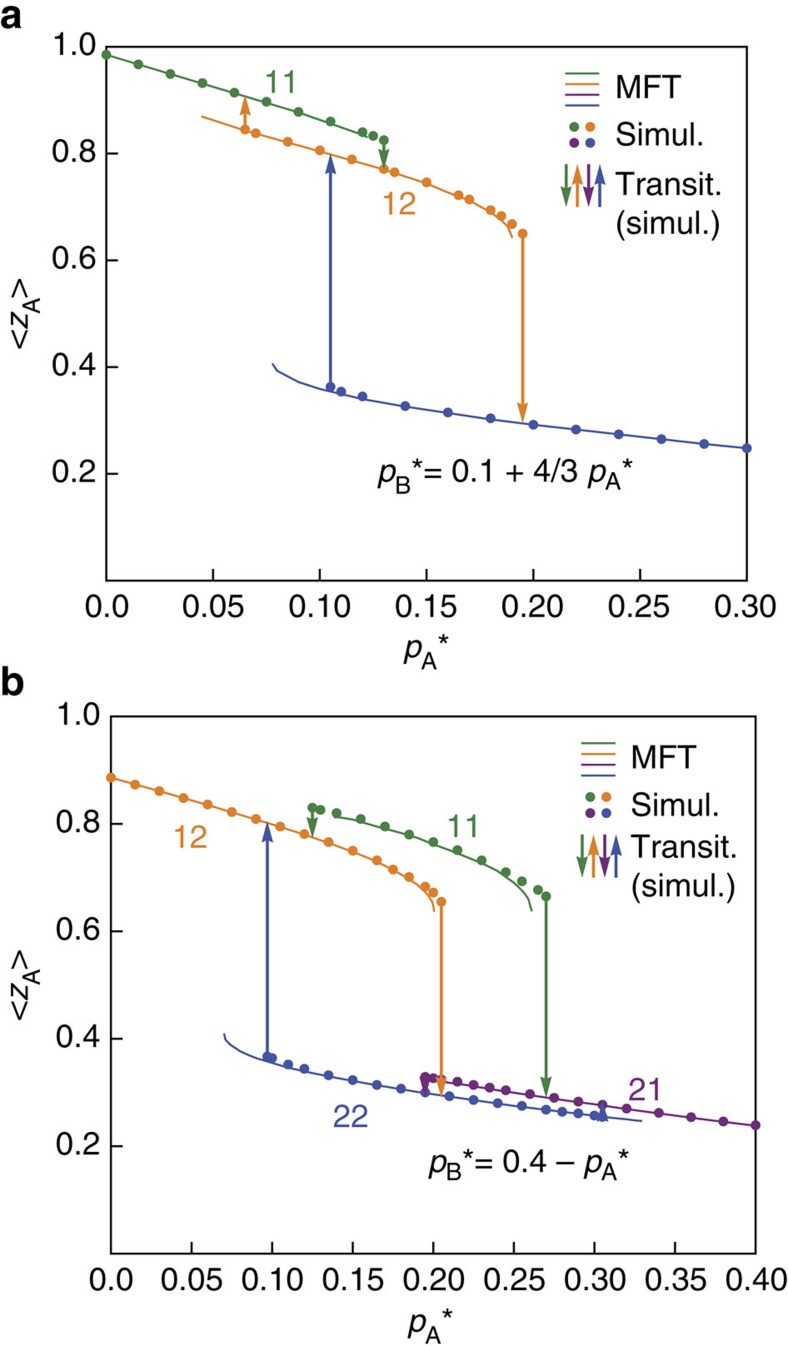
States with transitions and hysteresis loops for two activity profiles. (**a**) Activity *z*_A_ of network A, as measured in simulations (dots) and predicted by MFT (solid lines), along the cross-section defined by the black dashed line in Fig. 3. Parameters 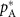 and 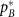 are changed, preserving the relation 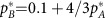. Transitions are denoted by arrows. (**b**) Same for the cross-section defined by 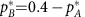 (red dashed line in Fig. 3). Here we obtain four states and six different transitions, giving rise to more complex hysteresis loops. Network parameters in all cases are (*k*=16, *m*=8).

We next can examine the activity profile for various cross-sections in the phase diagram. In Fig. 3 we choose two representative cross-sections (dashed straight lines) to measure activity *z*_A_=1−*a*_A_ as 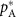 and 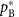 change. The black dashed line is defined by the equation 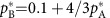 and the red dashed line by 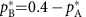. Figure 4a shows the activity measured in simulations of network A as we move along the black dashed line, changing both 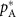 and 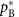, and preserving the relation 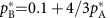. We perform simulations for various initial conditions and find (Fig. 4a) three different states denoted by green, orange and blue colours, which we identify as 11, 12 and 22 states, respectively. We find four different transitions: 11→12, 12→22, 12→11 and 22→12. The solid lines show the MFT prediction (equations (1) and (2)) for the activity of network A. The good agreement shows that the MFT correctly captures all the properties of the system. We note that qualitative agreement between the MFT and the simulations is better for higher values of *k*, because for higher *k* the fluctuations are smaller, which improves the accuracy of the MFT. Figure 4b shows the activity when moving along the red dashed line. Here we obtain four states and six different transitions.

The phase diagram of a system of *n*=2 interacting networks (Fig. 3) is much richer than the phase diagram of a single network with damage and recovery^12^. The analytical results we presented here for *n*=2 can be generalized to *n* interacting networks in any topological configuration, although as *n* increases they become increasingly difficult to visualize. In general, a system with *n* interacting networks can have up to 2^*n*^ physical states.

### The problem of optimal repairing

Knowing and understanding the phase diagram of interacting networks enable us to answer some fundamental and practical questions. A partially or completely collapsed system of *n*≥2 interacting networks in which some of them are in the low activity state is a scenario common in medicine, for example, when diseases or traumas affect the human body and a few organs are simultaneously damaged and need to be treated, and the interaction between the organs is critical. It is also common in economics, when two or more coupled sectors of the economy^18^ experience simultaneous problems, or when a few geographical clusters of countries experience economic difficulties. The practical question that arises is: what is the most efficient strategy to repair such a system? Many approaches are possible if resources are unlimited, but this is usually not the case and we would like to minimize the resources that we spend in the repairing process.

For simplicity, consider two interacting networks, both damaged (low activity). Is repairing both networks simultaneously the more efficient approach, or repairing them one after the other? What is the minimum amount of repair needed to make the system fully functional again? In other words, what is the minimum number of nodes we need to repair, to bring the system to the functional 11 (‘up–up') state, and how do we allocate repairs between the two networks? An optimal repairing strategy is essential when resources needed for repairing are limited or very expensive, when the time to repair the system is limited, or when the damage is still progressing through the system, threatening further collapse, and a quick and efficient intervention is needed.

We show below that this problem is equivalent to finding the minimum Manhattan distance between the point in the phase diagram where the damaged system is currently situated and the recovery transition lines to the 11 region. The Manhattan distance between two points is defined as the sum of absolute horizontal and vertical components of the vector connecting the points, with defined vertical and horizontal directions. It is a driving distance between two points in a rectangular grid of streets and avenues. In our phase diagram, it is equal to 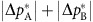. It turns out that two triple points of the phase diagram play a very important role in this fundamental problem. We find that these special points have a direct practical meaning and are not just a topological or thermodynamic curiosity.

To show this, we start by making some simplifying but reasonable assumptions. First, we assume that only internal failures can be repaired by human hands, as these failures are physical faults in nodes (any external and dependency failures and recoveries are ‘environmental', and are a spontaneous recognition of the changing neighbourhood of a node). We mentioned above that the parameters 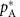 and 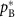 correspond to fractions of internally failed nodes in networks A and B, respectively. This implies that the number of internally failed nodes repaired in, say, network A, is directly proportional to the change of 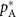. Hence, repairing nodes in networks A and B means decreasing 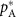 or 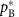. We also assume that these repairs are done fast enough that there is only a small probability that the newly repaired nodes will internally fail again before the repair process is completed. The total number of repaired nodes is therefore 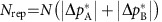 and it is proportional to the Manhattan distance between the starting and final point in the phase diagram.

**Figure 5:**
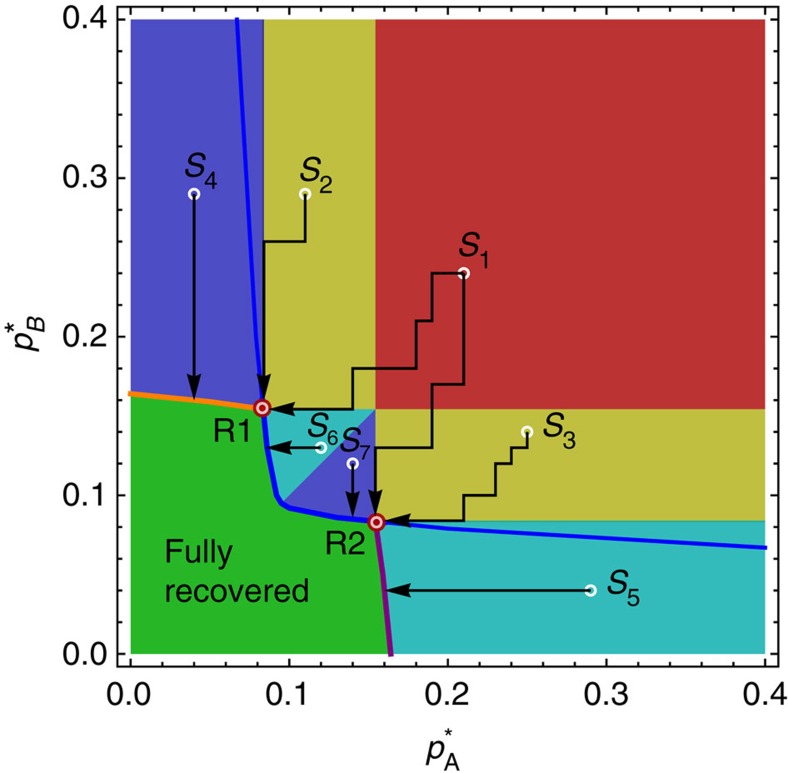
Optimal repairing strategies. The optimal repairing procedure (least expensive in terms of the number of individual node repairs) depends on the initial condition of the collapsed system. The total cost of repair is 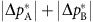 and the problem of optimal repairing translates into finding the minimal Manhattan distance from the point (in the phase diagram) where the collapsed system is initially situated (*S*_i_) to the nearest border of the green region where it becomes fully functional. For a system having the initial condition within the red section (for example, point *S*_1_), there are two solutions: it is equally optimal to reach any of the two triple points R1 and R2 by decreasing 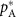 and 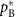. For the systems starting in the yellow regions, it is optimal to reach only one triple point, R1, for the sector containing point *S*_2_, or R2 for the sector containing point *S*_3_. Starting in the dark blue regions it is optimal to decrease 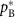 only, that is, repairing only network B. Similarly, in the light blue regions it is optimal to decrease 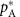 only. Triple points play a crucial role when both networks are initially significantly damaged (red and yellow regions).

To optimize repairing we need to minimize this metric. Figure 5 shows the solution to the minimization problem and a detailed discussion is provided in the Methods section. The different colours in Fig. 5 correspond to the different optimal repair strategies, which depend on the failure state of the system. If the system is initially at point *S*_1_, both networks are in a low activity state, that is, they are non-functional. Our goal is to decrease 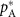 and 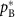, and arrive to the region where the system is fully recovered (the green region) by performing a minimal number of repairs, that is, minimal *N*_rep_. We find that for any point in the red region there are actually two closest points in the green region, at an equal Manhattan distance away from the red region point. These two points are the triple points R1 and R2 shown in Fig. 5, which also correspond to the triple points in Fig. 2b. Although R1 may be closer to point A than R2 by Euclidian distance, the Manhattan distance is the same. Thus, two equally good repairing strategies are available. One involves allocating more node repairs to network A and the other allocating more repairs to network B. For the yellow regions (points *S*_2_ and *S*_3_), the closest points by Manhattan distance are R1 (for point *S*_2_) or R2 (for point *S*_3_). Here, only one triple point represents the optimal solution. It is noteworthy that the path samples in Fig. 5 are ‘zig-zag' in shape (to highlight that we are minimizing 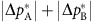); however, even when a diagonal path (direct straight line) to a triple point is used, the Manhattan distance is the same. For the dark blue regions (points *S*_4_ and *S*_7_), the optimal strategy is to decrease 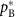 only, until the system is recovered. Similarly, for the light blue regions (points *S*_5_ and *S*_6_), the optimal strategy is to decrease only 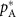.

From our optimal repairing strategy analysis we find that the order of repair (the specific path taken between the initial point and final point) does not affect the final result. Minimizing the Manhattan distance only determines the optimal destination point. Therefore, there is actually a set of paths corresponding to equally optimal repairing processes.

### States and transitions in real-world networks

**Figure 6:**
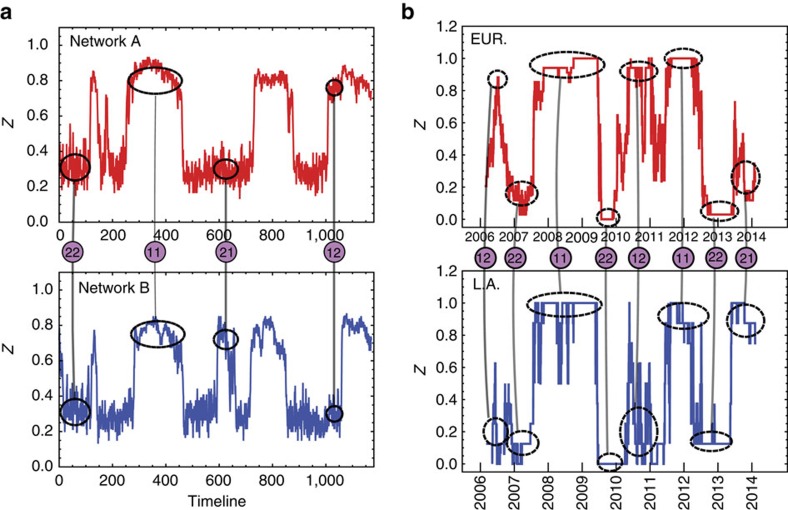
Collective dynamics in simulated and real interacting networks. (**a**) Simulation of the networks' dynamics, activity versus time, for *N*=100 and failure parameters 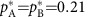, *r*_A_=*r*_B_=0.60 and *r*_d_=0.15 shows the switching of the system between four different states. We can easily identify four collective states: 11, 22, 12 and 21. (**b**) Dynamics of two CDS geographical networks consisting of 17 European and 8 Latin American countries, showing very similar behaviour: individual networks switching between well-defined high-activity and low-activity states, as well as correlated collective behaviour of the two networks in interaction. We identify collective states 11, 22, 12 and 21, and mark them with connected black ovals. It is noteworthy that, as the CDS value grows with risk, a higher activity in a CDS network corresponds to bad economic news.

In relatively small networks (*N*≈10–1,000) fluctuations are very large. Thus, in small network systems exhibiting multistability it is possible to observe phase flipping^12^,^15^,^33^ between different states. Figure 6a shows the fraction of active nodes for both networks, in time, for a symmetric choice of parameters, 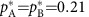, *r*_A_=*r*_B_=0.60 and *r*_d_=0.15, when each network has only *N*=100 nodes. Large fluctuations cause the system to jump between the different states allowed for this set of parameters. It is noteworthy that interdependent links cause the two networks to have partially dependent and correlated dynamics. Very often a transition in one network triggers a transition in the other. In Fig. 6a we can identify examples of all four global states: 22, 11, 21 and 12. For example, at time *t*≈400 both networks are in the high activity state (11), whereas at *t*≈620 network A is in the low activity and network B in the high activity state (21).

As many real-world interacting network systems have a small number of nodes, in those systems we can potentially uncover dynamics similar to what we observe in our model networks. As an example of a real system, we investigate the interacting sovereign 5-year CDS system, consisting of 25 European Union (EU) and Latin American countries (see Methods for the full list of countries) that began to issue the CDSs from 2005. We divide countries into two groups on a geographical basis: 8 countries belong to the Latin American group and 17 belong to the European Union group. Sovereign CDSs are financial instruments, for which the value reflects the probability that the reference country will default on its debts. Each country has a CDS value assigned and this value changes in time reflecting the economic news about this country and the perceived risk of default, which results in a time series that we can observe. CDSs are highly sensitive to important economic news, positive or negative. There is also a significant contagion and influence between the countries, especially between those with strong economic ties, which is reflected in the correlation between their CDSs. These characteristics make the CDS signals a candidate for modelling using our interacting network approach.

We can draw a parallel between the CDS system and our model network if we assume that each country (with its associated CDS signal) can be represented as a node, which has connections (links) to other countries within its own geographical region, as well as ties with countries from another continent. In this case, we might expect that random and independent bad (or good) economic news appearing in any given country have behaviour similar to random internal processes in nodes in our artificial model (random internal failures/recoveries). When economic problems in one country propagate to a neighbouring country within the same geographical region, the process resembles the external failures in our artificial model, whereas interaction between countries from different continents may be modelled by the interdependent links from our network model. For the CDS network system we also suppose that the fractional definition for the threshold (*m*_frac_) is somewhat more natural then the absolute definition, as it is less dependent on the country size or its importance, that is, the number of links a country has to other countries.

**Figure 7:**
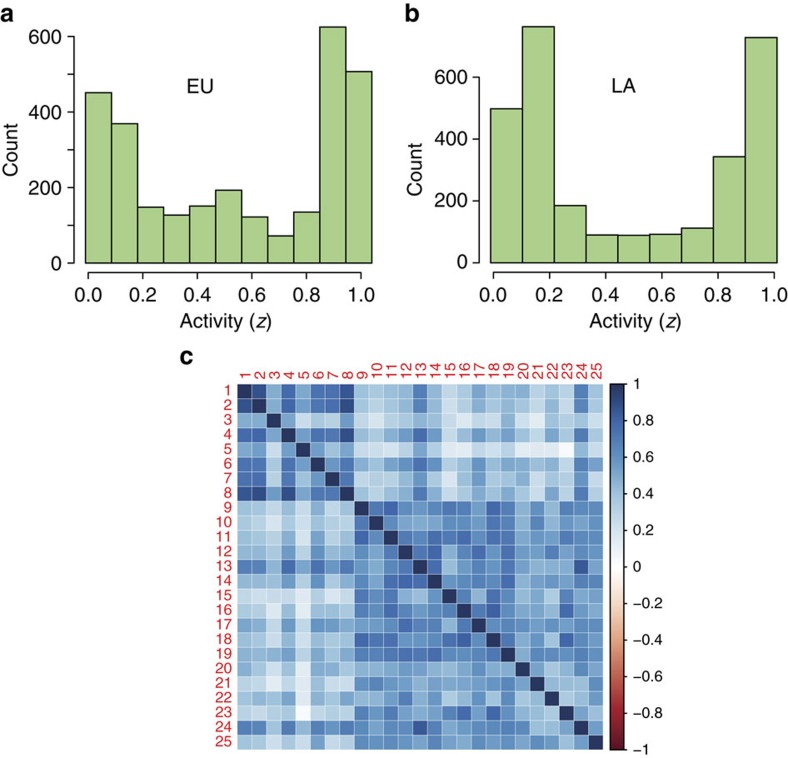
Real network dynamics. (**a**) Activity density plot for the EU network reveals bimodality, an indication of the existence of two states. (**b**) Same for the LA network. (**c**) Correlation matrix of binary CDS signals with the EU (1–8) and LA (9–25) block. Separation into blocks reinforces our initial decision to sort the countries by geographic location.

We study the international CDS system during the period between June 2005, the earliest date when CDSs traded for all countries, and February 2014. We apply the network model to it as follows. We represent each country with one node that can have two states: active or failed. As the raw CDS values are continuous by nature and our model uses binary node states (up or down), we perform a trend mapping procedure to form a binary signal (0 or 1) for each country. In particular, for each time *t*, we consider the interval [*t*−252, *t*] of 252 business days (the usual number of business days in a year). If the CDS value of a country has a net increase during that period, we consider the node of the country to be active at *t* (state=1). If it does not, it is inactive (state=0). Having individual binary signals for each country, we can calculate the average activity 0≤*z*(*t*)≤1 for both EU and Latin American networks. The resulting time series for EU and LA activities are shown in Fig. 6b. First, we note that the two geographical networks spend most of time having either a significantly high activity or significantly low activity (that is, there is an indication for two well-defined single-network states). We confirm this by measuring the frequency distribution of network activities (Fig. 7a,b), which exhibit a strong bimodality in *z*. The CDS network system in Fig. 6b shows rapid transitions between the high and low activity states, much similar to the artificial network system in Fig. 6a. Figure 7c shows the calculated correlations between binary signals of pairs of individual nodes. The correlation matrix reveals two strongly correlated blocks, which we identify as Latin American block (numbers 1–8) and EU block (numbers 9–25).

In Fig. 6b, we also observe that the two networks sometimes make transitions simultaneously, but not always. This behaviour also resembles the behaviour observed in the artificial networks in Fig. 6a.

Finally, we find that it is possible to estimate numerical values for all the model parameters of this real system (internal 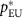, 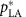; external *m*_frac,EU_, *m*_frac,LA_, *r*_EU_, *r*_LA_; and interdependent *r*_d_) from the data. The basic idea is that for each parameter we identify an observation experiment in which this particular parameter dominates, enabling us to effectively isolate individual parameters from the noise of many others. For example, when both networks (EU and LA) are in the high activity phase, most of the failures are in fact internal failures. This allows us to almost directly estimate 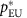 and 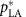 from Fig. 6b, by observing 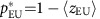 and 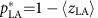. External failures are most significant when a network is in a low activity state. Interdependent parameter *r*_d_ can be estimated by studying the correlation between *z*_EU_(*t*) and *z*_LA_(*t*), as this is an increasing function of *r*_d_. Threshold parameters *m*_frac,EU_ and *m*_frac,LA_ can be estimated by exploiting the fact that they most significantly determine the fraction of time that each network spends in the high, or low, activity states. We describe in detail our procedures for numerically estimating these model parameters in the Supplementary Methods. The procedure for estimating some of these parameters is also illustrated in Supplementary Fig. 1. Numerical results for the parameter estimates are presented in Supplementary Table 1. Our dynamical network model also independently predicts that the typical fluctuation size of *z*(*t*) is not uniform for all values of *z*, but has a spike around 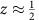. We observe this phenomenon in both our simulations and the real network dynamics (Supplementary Fig. 2).

## Discussion

Interacting networks appear across many disciplines, from medicine, physics, biology and ecology, finance and economics, to infrastructure^34^,^35^ (refs 34,35). We propose a generic model that captures some of the most common processes found in real interacting networks—node failure, systemic damage propagation and node recovery. We report several intriguing results. Our solution of the model produces a rich phase diagram with a number of tipping points (critical points, triple points and transition lines). Using the phase diagram, we solve a fundamental problem of the optimal repairing strategy for a damaged system consisting of interconnected networks. Solving this problem enables us to determine the minimum set of node repairs required to repair a failed interconnected network system; thus, it becomes fully functional again. Remarkably, we find that the triple points from our phase diagram play the dominant role in constructing the optimal repairing strategy in damaged interacting systems. This implies that triple points are not only a thermodynamic or topological curiosity, but they have a very direct practical meaning and application, specifically in constructing repairing strategies in interacting systems. The problem of functional repair using minimal intervention is relevant in medicine, economics and other disciplines, when repairs are invasive or expensive. Finally, we apply our model to a selected real system: interconnected networks of CDSs, for two interconnected groups of countries. We propose a methodology to measure all of the model parameters in a real system, by using observational experiments in which a particular parameter dominates the behaviour of the system (see Supplementary Information).

## Methods

### Damage conductivity parameters

Parameters *r*_A_ and *r*_B_ are introduced, because they describe how easily the damage is spread through the network. When *r*=0 there is no damage spread between the nodes, and when *r*=1 there is perfect damage conduction. Assuming that external failures occur with certainty would mean fixing *r* to be equal to 1. In the case of a single network with recovery, it has been shown^12^ that many important phenomena (for example, spontaneous recovery) are lost when *r*=1. The most interesting parts of the phase diagram are in fact where *r* is far from 1.

It is noteworthy that our choice of the value for *r*_d_ is quite limited. If *r*_d_ is too large, we find that the damage spreads through dependency links extremely efficiently and the only possible stable state is total system collapse. The extreme vulnerability of interdependent networks is well known^2^,^22^. As there is always at least one functional stable state in biological or man-made systems, total system collapse as the only stable state is not realistic. Thus, we need the *r*_d_ parameter to ‘soften' the dependency links^22^ and allow a more realistic behaviour and its value should not be close to 1.

### Mean field theory

Fractions *a*_A_ and *a*_B_ denote the fraction of nodes that are failed due to any of the three types of failures: internal (I), external (E) or dependency failure (D). We denote the probabilities that a node at a time of observation experiences a failure of I, E or D type as *P*(I), *P*(E) and *P*(D), respectively. As a first approximation, we assume that these failures are mutually independent events. Considering network A first, we write an expression for the probability *a*_A,*k*_ that a node of degree *k* in network A is failed. The node can fail due to I, E or D events, or to a combination of them. Using the inclusion–exclusion principle for independent events, we write


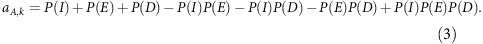


Next, we separately calculate *P*(I), *P*(E) and *P*(D).

*P*(I) is also the average fraction of internally failed nodes in a network, as internal failures are independent events. This is a Poisson process on individual nodes^12^,^36^ and therefore 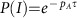. As parameters *p*_A_ and 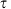 come in this expression as a product, we can replace them with a single parameter, 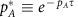, which is bounded and also has the property 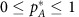 and thus 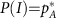 for network A.

Next, we calculate *P*(E), the probability that a randomly chosen node with degree *k* has externally failed. Focusing once again on network A, without a loss of generality, we let *F*(*k*) be the probability that a node of degree *k* in network A is located in a critically damaged neighbourhood (where fewer than *m*+1 nodes are active). By definition, the time-averaged fraction of failed nodes (for any reason) in network A is 0≤*a*_A_≤1. In a mean-field approximation, this is also the average probability that a randomly chosen node in that network has failed. Using combinatorics, we obtain 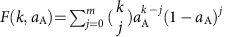 (ref. 12). The probability that a node of degree *k* in network A has externally failed is then *P*(E)=*r*_A_*F*(*k*, *a*_A_). An analogous result is valid for network B.

Finally, we calculate *P*(D), the probability that a node has failed due to the failure of its dependent counterpart node in the other network. For network A, this probability is equal to the product of parameter *r*_d_ and the probability that a counterpart node in B has failed: *P*(*D*)=*r*_d_*a*_B_. In network B by analogy, this probability is equal to *r*_d_*a*_A_.

Writing equation (3) for both networks and inserting the results for *P*(I), *P*(E) and *P*(D) after summing over all *k* (and noting 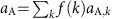 and 
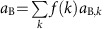
), we get a system of two coupled equations that describes the system of networks,


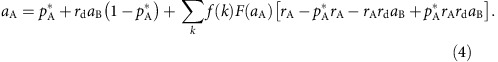



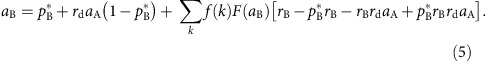


### Additional phase diagrams

**Figure 8:**
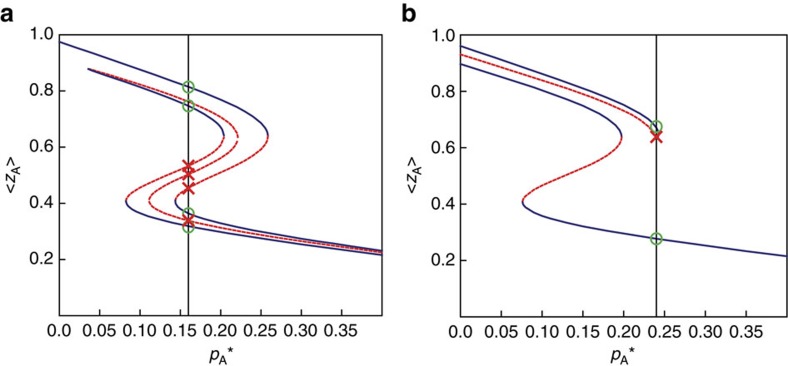
Activity of network A versus internal failure rate. (**a**) Activity *z*_A_=1−*a*_A_ obtained by solving equations (1) and (2), for a range of 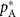 values, in a system of two interdependent networks (*k*=16, *m*=8). Blue lines correspond to stable physical states and red dotted lines represent unstable solutions. In Fig. 8a, the same parameters as in Fig. 1a are used, except 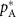, which is not fixed but varied. When 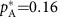 (vertical black line), the corresponding values on the blue solid lines (green circles) match the graphical solutions in Fig. 1a (also green circles). (**b**) An analogous relationship holds between Figs 1b and 8b, in which case two stable states exist.

Figure 8a shows the collection of stable solutions (solid blue lines) and unstable solutions (dashed red lines) for the activity *z*_A_=1−*a*_A_ of network A, with parameter values as used in Fig. 1a, but for a range of different values of 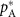. The solid black line indicates 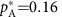, the value of 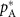 used in Fig. 1a. Green circles in this figure correspond to the stable states found in Fig. 1a and red crosses correspond to the unstable solutions for *z*_A_ form Fig. 1a. Figure 8b shows an analogous phase diagram for the parameters with values as in Fig. 8a, again for a range of 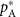.

### Forbidden transitions

Transition lines for 12→21 and 21→12 do not appear in our phase diagram and it is quite easy to understand why. Let us assume that the transition line for 12→21 does exist. To obtain that transition, the idea would be to simultaneously increase 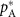 and decrease 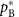 (that is, increase the damage in one part of the system and decrease in another part). Suppose we are in phase 12 and infinitesimally close to the supposed transition line. Considering the local geometry of this line, we may be able to observe its angle with respect to the 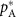 axis. If a transition occurs when increasing 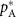 and decreasing 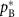, the tangent on the supposed line would have an angle of 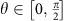. From here, it follows that by increasing 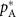 only, while keeping 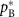 constant, we would also make a transition (cross the transition line). The only other possibility would be that we were moving along the transition line, but this is easy to disprove because it would imply that the transition does not depend on 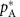. If increasing 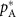 only causes a transition, the transition must end in state 22 and not in 21. This is because if we only increase 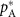, we increase damage to both network A (directly) and network B (indirectly, through the interdependent links).

### Geometry of the Manhattan distance minimization problem

**Figure 9:**
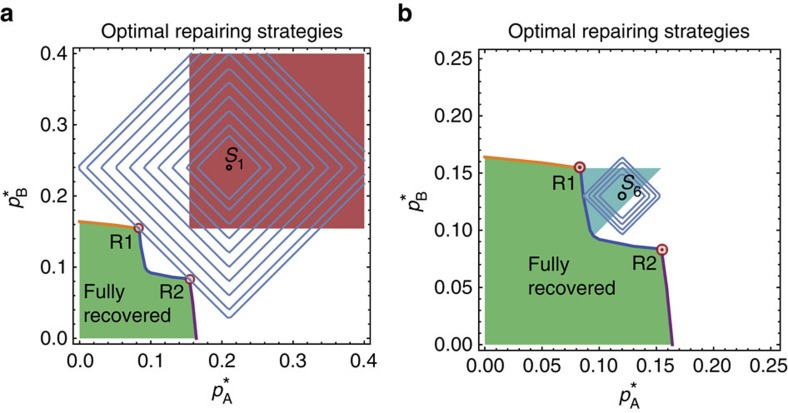
Minimum Manhattan distance problem in two examples. (**a**) Finding the minimum Manhattan distance between point *S*_1_ in the red sector and the green region where the system is fully functional. Equidistant curves are plotted in grey and form a ‘diamond' shape. The largest ‘diamond', barely touching the green region and having its centre at point *S*_1_, suggests there are two equally optimal solutions to the minimization problem: points R1 and R2. (**b**) The same geometrical construction for point *S*_6_ in the light blue region, suggests a unique solution: decreasing 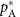.

The optimal strategies shown in different colours in Fig. 5 are derived from the geometrical reasoning shown in Fig. 9. Figure 9a shows a plot of a series of curves consisting of points at identical Manhattan distances from point *S*_1_ (equidistant curves). They produce a ‘diamond' shape, and the minimal Manhattan distance between point *S*_1_ and the green region translates into the task of ‘fitting' the diamond so that it just touches the green region and its centre is at *S*_1_. The diamond in Fig. 9a touches the green region at two points—triple points, which are the solution to the minimization problem. Figure 9b shows the solution for point *S*_6_ in the light blue region. Here the solution suggests a different strategy—decreasing only 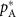.

### Credit default swaps

Figure 6b shows an analysis of 5-year sovereign debt CDSs for a set of European countries: France, Germany, Italy, Spain, Portugal, Belgium, Austria, Denmark, Sweden, Greece, Ukraine, Hungary, Poland, Croatia, Slovenia, Romania, Bulgaria and Slovakia. This is the set of European countries that had a sovereign debt CDS in 2005. The set of Latin American countries we analyse consists of Brazil, Colombia, Argentina, Mexico, Venezuela, Chile, Peru and Panama. A CDS is typically used to transfer the credit exposure of fixed income products from one party to another. The buyer of the CDS is then obligated to make periodic payments to the seller of the CDS until the swap contract matures. In return, the seller of the CDS agrees to compensate (pay off) the seller who holds this third party debt if this (third party) defaults on the issued debt.

A CDS is, in effect, an insurance against non-payment of a debt owed by a third party. The buyer of a CDS does not have to hold the debt of the third party but can speculate on the possibility that the third party will indeed default and the buyer can purchase the CDS for this speculative purpose. CDSs were developed in the 1990s and, given their simple structure and flexible conditions, they are now a major part of the credit derivative activity in the OTC market used to hedge credit risk. One of the most important aspects of a CDS is the definition of the ‘credit event' that triggers the CDS. These events include bankruptcy, obligation acceleration, obligation default, failure to pay, repudiation (moratorium) and restructuring. In the case of the sovereign bond market, the last three are typically included in the contracts. CDSs are used by investors to hedge exposure to a fixed income instrument, to speculate on likelihood of a third party (reference asset) default, or to invest in foreign country credit without currency exposure.

## Additional information

**How to cite this article:** Majdandzic, A. *et al.* Multiple tipping points and optimal repairing in interacting networks. *Nat. Commun.* 7:10850 doi: 10.1038/ncomms10850 (2016).

## Supplementary Material

Supplementary Information Supplementary Figures 1-2, Supplementary Table 1 and Supplementary Methods.
