# Supplementary material for: Multiple tipping points and optimal repairing in interacting networks
Source: Nat Commun. 2016 Mar 1;7:10850. doi: 10.1038/ncomms10850 (PMC4773515; doi:10.1038/ncomms10850)
Supplement: Supplementary Information — Supplementary Figures 1-2, Supplementary Table 1 and Supplementary Methods. [file ncomms10850-s1.pdf]

I. SUPPLEMENTARY FIGURES

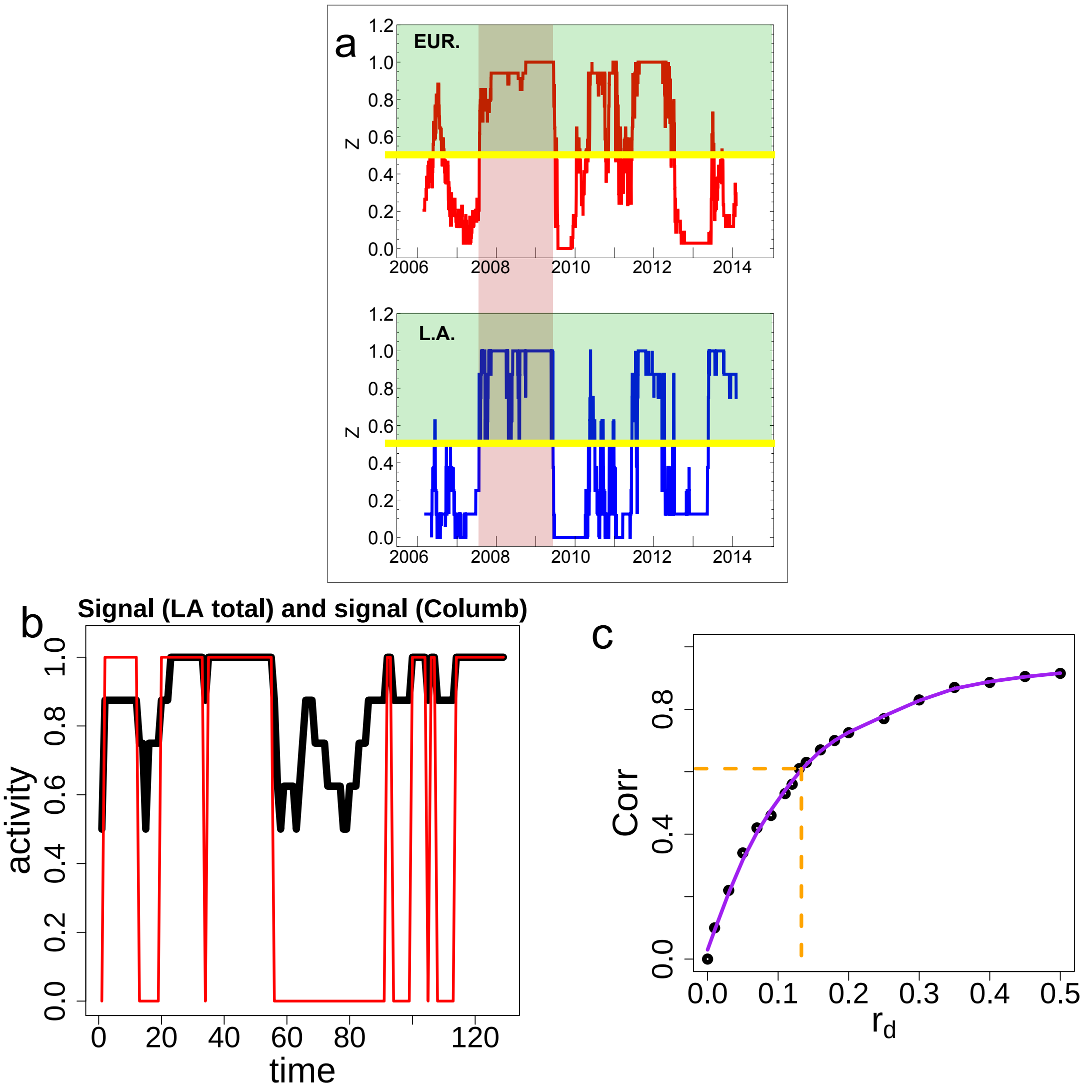

FIG. 1: (SUPPLEMENTARY FIGURE 1):
Quantifying the CDS network and estimating model parameters.
a Estimating p_A^* and p_B^* by observing intervals in which both networks have high activity ($z \geq \frac{1}{2}$). The region of interest (above $z = \frac{1}{2}$, yellow line) is colored in green, with an example interval in red. **b** Dynamics of an individual binary signal (Columbia), when the LA network is in the high activity state ($z_{LA} \geq \frac{1}{2}$). **c** Matching the value of r_d for $Corr = 0.61$: correlation between the signals of two artificial networks with $N_{\mathrm{A}} = N_{\mathrm{EU}} = 17$ and $N_{\mathrm{B}} = N_{\mathrm{LA}} = 8$ nodes, for different values of r_d, with other parameters fixed to the values from $\langle k \rangle = 5$ row in Table 1.

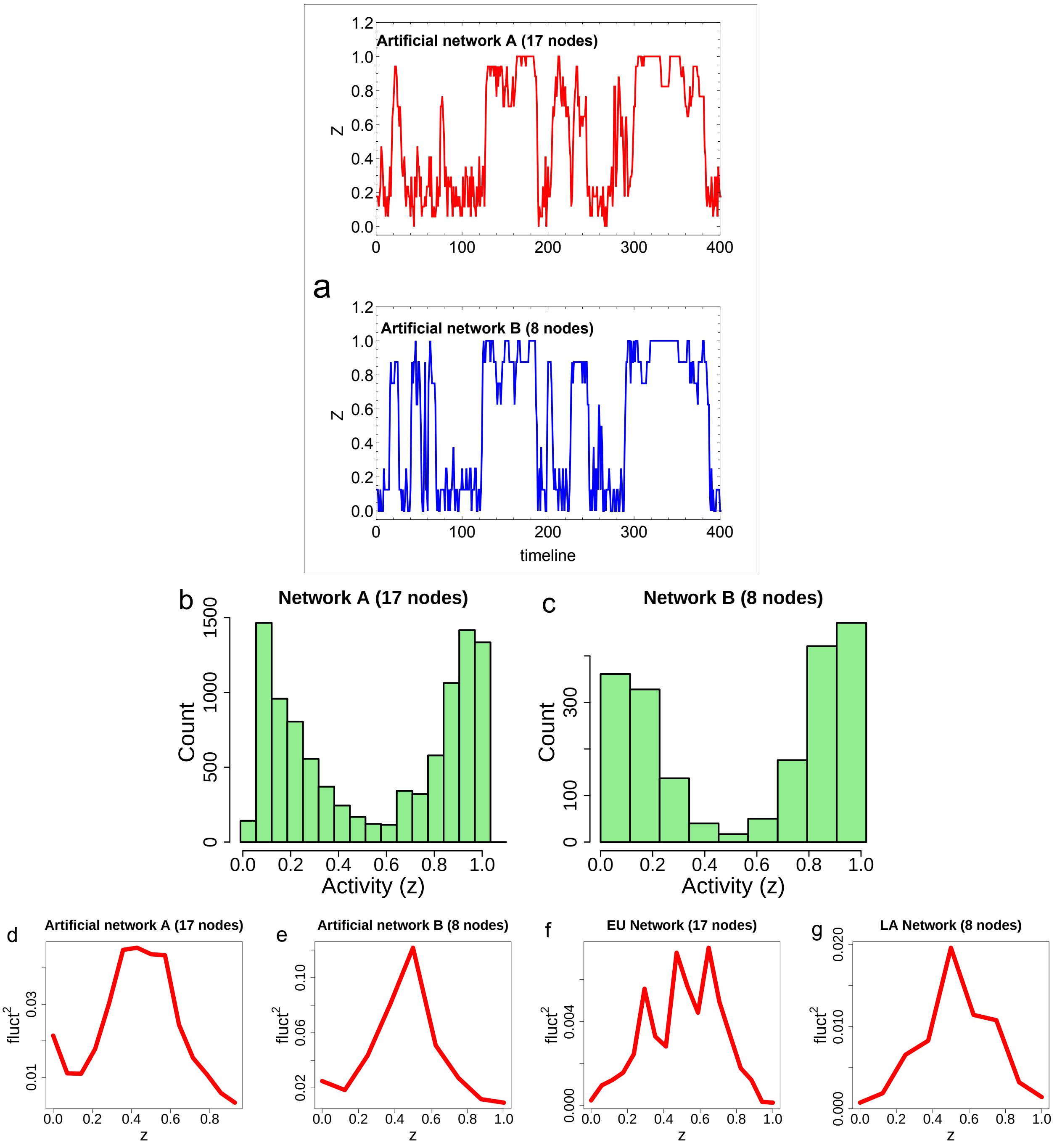

FIG. 2: (SUPPLEMENTARY FIGURE 2):
Analysis of network dynamics. **a** Two model (artificial) networks: Typical time evolution of activity. **b–c** Activity density plot for two model networks reveals bimodality. **d–g** Fluctuation size peaks around $z \approx 0.5$ for model networks A (**d**) and B (**e**). A very similar pattern is found in real networks, EU (**f**) and LA (**g**).

II. SUPPLEMENTARY TABLES

$\langle k\rangle$	$m_{frac,EU}$	$m_{frac,LA}$	r_EU	r_LA	r_d
3	0.55	0.43-0.49	0.78	0.87	0.16
4	0.55	0.43-0.49	0.79	0.87	0.14
5	0.57	0.43-0.49	0.79	0.87	0.13
6	0.57	0.43-0.49	0.79	0.87	0.11
7	0.57	0.43-0.49	0.79	0.87	0.10

TABLE I: (SUPPLEMENTARY TABLE 1):
Numerical estimates for the model parameters. Using this "isolation method" we find limits on the numerical values of the model parameters. These observation experiments allow us to gauge the strength of various effects in real world systems. Here we confirm experimental accessibility of all of the model parameters. Note that values for r_{d} are rather small - this is expected and discussed in Methods.

III. SUPPLEMENTARY METHODS

Applying the model to the CDS network: Outline

There are two steps in applying our model to the system of binary CDS signals: 1) *building the network* (choosing the rules for connecting the nodes), and 2) *estimating model parameters* (for internal failures: p^*_A, p^*_B, for external failures: $m_{frac,A}$, $m_{frac,B}$, r_A, r_B, and for the interdependent interaction: r_d) which are the core of our model. We first make reasonable assumptions to model the structure of the links. We then show that we are capable of estimating – based on the real records – all of the model parameters, by analyzing the situations in which one of these parameters dominates the dynamics of the system.

Building the CDS network

Because the CDS system is a symbolic network, we have a certain freedom in modelling the links connecting the countries. First, we make few assumptions. We assume that LA and EU countries are each represented by a network, one with $N_{\mathrm{LA}} = 8$ nodes and the other with $N_{\mathrm{EU}} = 17$ nodes. We make a simplifying assumption that both have the same average degree $\langle k\rangle$, which we do not fix: we vary it and investigate how it affects the estimates of dynamics parameters. We assume a probabilistic rule for the links: the probability that there is a link between two nodes within the same network is *proportional to the correlation coefficient* between the nodes taken from the correlation matrix in Fig. 7c. Because the total number of links per network is fixed to $\langle k\rangle \frac{N}{2}$, and the links are probabilistic, this results in many possible physical realizations for each network. We run simulations for a large sample of such network realizations, with the same parameters, and average over the ensemble. During each simulation, the links do not change. For the interdependent links we assume that every node in both the LA and EU networks has at least one interdependent link, but some nodes can have more than one (because $N_{LA} \neq N_{EU}$ it is not possible to have one-to-one interdependency). Here we also apply the probabilistic rule: the probability that there is an interdependent link between a node in EU and a node in LA is proportional to the associated matrix element in the correlation matrix in Fig. 7c. There is a degree of freedom for the total number (L) of interdependent links between the two networks: we do not fix this value but investigate how various quantities depend on the range of reasonable values of L.

Measuring and estimating model parameters

We find that it is possible to estimate from the data all the model parameters (internal p^*_{EU}, p^*_{LA}, external $m_{\mathrm{frac,EU}}$, $m_{\mathrm{frac,LA}}$, r_{EU}, r_{LA}, interdependent r_d), using quite simple and reasonable arguments. For each parameter, we identify an *observation experiment*, a part of the dynamics, or a phenomenon, in which this particular parameter dominates. This method enables us to effectively isolate individual parameters from the noise of many others. Below we outline our procedure.

Estimating internal parameters (p^*_{EU}, p^*_{LA}): observing high activity states.

When both networks (EU and LA) are in the high activity phase, most of the failures are in fact *internal failures*. In Fig. 6a for example, only about 3% of the failures observed during the high activity are external failures, because there is a small chance for having a critically damaged neighborhood when z is very high. The contribution from

interdependent failures when both networks have high activity is also rather small (of the order of $(1-z)r_d$, where both $(1-z)$ and r_d are small). This allows us to almost directly estimate p^*_{EU} and p^*_{LA} from the real records, because with only internal failures present we know that $\langle z\rangle = 1-p^*$, so $p^*_{\mathrm{EU}} = 1-\langle z_{\mathrm{EU}}\rangle$ and $p^*_{\mathrm{LA}} = 1-\langle z_{\mathrm{LA}}\rangle$. By measuring the average $\langle z_{\mathrm{EU}}\rangle$ and $\langle z_{\mathrm{LA}}\rangle$ for the times when both networks are highly active (such as when $z_{\mathrm{EU}} > 1/2$ and $z_{\mathrm{LA}} > 1/2$ - an example of a time interval with that condition is colored in red in SuppFig. 1a), we get estimates $p^*_{\mathrm{EU}} = 0.07 \pm 0.01$ and $p^*_{\mathrm{LA}} = 0.11 \pm 0.02$.

Alternative measurement of the internal parameters: micro-dynamics.

Alternatively, by observing the network on the micro level, i.e. observing the dynamics of individual nodes, it is also possible to measure the average internal time of recovery, τ_{EU} and τ_{LA}, the crude failure rates p_{EU} and p_{LA}, and to verify the previously measured p^*_{EU} and p^*_{LA} in an alternative way. Supplementary Figure 1b shows an example of the Columbia signal (red) for a fraction of time between November 2006 and March 2007, when the activity of both LA and EU networks was in the upper half ($z_{\mathrm{EU}} > 1/2$, $z_{\mathrm{LA}} > 1/2$). By measuring *how long* a country (node) stays in the 0 state each time it fails under these conditions, and measuring *how often* it fails, we can estimate its mean recovery time and the crude failure rate. We find the average values for the EU countries to be $\tau_{\mathrm{EU}} = 13 \pm 2$, $p_{\mathrm{EU}} = (3.0 \pm 0.5) * 10^{-3}$ and $p^*_{\mathrm{EU}} = 0.07 \pm 0.01$ (same as before). For LA countries we find the average values of $\tau_{\mathrm{LA}} = 9 \pm 1$, $p_{\mathrm{LA}} = (7.6 \pm 0.8) * 10^{-}3$ and $p^*_{\mathrm{LA}} = 0.11 \pm 0.02$ (same as before). Note that because for any nonlinear function f and random variable x, the expectation $\mathrm{E}(f(x)) \neq f(\mathrm{E}(x))$, we have $\langle p^*\rangle \neq 1 - exp(-\langle p\rangle\langle\tau\rangle)$.

Estimating the external parameters r_{EU} and r_{LA}: observing low activity states.

When both networks are in the low activity state (low values of z for EU and LA), external failures dominate. Internal failures are present all the time, but they are known as we have already estimated the internal failure parameters. To get a first estimate of r_{EU} and r_{LA}, we will neglect the interdependent failures temporarily, which are also present but much weaker then external failures, and assume that the networks are weakly coupled (when we later estimate the value of r_d, we will get a small correction for the values of r_{EU} and r_{LA}). Also, luckily, for low values of z, and if m_{frac} has a reasonable value (not too close to 0, and not too close to 1, which is always satisfied by requiring the existence of two states per network - values of m_{frac} too close to 0 or 1 lead to single states), nearly every node has a critically damaged neighborhood, so the dependence on m_{frac} vanishes and in this case the value of $\langle z\rangle$ is almost independent on the threshold m_{frac}. Indeed, our simulations confirm that the positions of the network stable states (which in the thermodynamic limit coincide with $\langle z_{low}\rangle$ and $\langle z_{high}\rangle$) do not depend much on m_{frac}. The parameter m_{frac} however strongly determines the amount of time a network spends in the upper state, as opposed to the lower state, but it has little influence on the position of those states. Because of the structure of function $F(1-z; m_{frac}) \approx \theta(1-z-m_{frac})$ in Eq. (1) and (2), which behaves similarly to the Heaviside step function θ, the influence of m_{frac} on the dynamics is strongest when $1-z(t)$ is close to m_{frac}. However this is rare when the system is in the low or high activity states where it spends most of its time; medium values of z are usually achieved when the system makes a transition which lasts shortly and does not contribute much to $\langle z\rangle$. Appreciating these simplifications, the equation that connects the average fraction of failed nodes with the internal and external parameters becomes 1-$\langle z_i\rangle = {p_i}^* + r_i - {p_i}^* r_i$ (where i = {EU, LA}), with r_i as the only unknown. By measuring $\langle z_i\rangle$ in the low states ($z_{\mathrm{EU}} < 1/2$ & $z_{\mathrm{LA}} < 1/2$) and already knowing ${p_i}^*$, we obtain estimates $r_{\mathrm{EU}} = 0.81 \pm 0.03$ and $r_{\mathrm{LA}} = 0.88 \pm 0.03$.

Estimating the thresholds $m_{\mathrm{frac,EU}}$ and $m_{\mathrm{frac,LA}}$: visiting times

If we picture the two states per network (low and high) as a double well, then the parameter m_{frac} through the function $F(z; m_{frac})$ controls the position and the shape of the potential barrier between the wells, and m_{frac} dominates in determining the total fraction of time that each network spends in the upper, or lower, state. Higher values of m_{frac} lead to the network spending more time in the low activity state, and vice versa. This is another useful observation, which allows us to estimate the values of $m_{\mathrm{frac,EU}}$ and $m_{\mathrm{frac,LA}}$ from the real data. In Figure 6b, both networks spend approximately half of their time in the high activity state or $z \geq 1/2$ (precisely, EU - 53%, LA - 47%). By simulating decoupled ($r_d = 0$) EU and LA networks using previously measured parameters (p^*_{EU}, p^*_{LA}, τ_{EU}, τ_{LA}, r_{EU}, r_{LA}), and requiring that the networks spend roughly 50% of time in each state, we are able to get first estimates for the thresholds. We find that they slightly depend on the choice of $\langle k\rangle$, ranging from $m_{\mathrm{frac,EU}} = 0.57 \pm 0.02$ and $m_{\mathrm{frac,LA}} = 0.50 \pm 0.02$ for $\langle k\rangle = 3$, to $m_{\mathrm{frac,EU}} = 0.59 \pm 0.02$ and $m_{\mathrm{frac,LA}} = 0.50 \pm 0.02$ for $\langle k\rangle = 7$ ($\langle k\rangle$ is limited by the number of nodes in the smaller network to a maximum of 7). Later, after we estimate r_d, we will get small corrections for $m_{\mathrm{frac,EU}}$ and $m_{\mathrm{frac,LA}}$ by simulating the networks with a nonzero value of r_d.

Estimating r_d: correlation between networks EU and LA

The parameter r_d represents the interaction strength of the interdependent nodes in the two networks. If r_d were zero, the two networks would have perfectly independent dynamics. On the other extreme, if $r_d = 1$ we would expect the two networks to have extremely correlated dynamics. Thus, studying the correlation between $z_{\mathrm{EU}}(t)$ and $z_{\mathrm{LA}}(t)$ represents a natural way for estimating the interaction parameter r_d. The correlation between the two real signals in Fig. 6b has the value of 0.61. The idea for measuring r_d is straightforward: By simulating an artificial interacting network system, using our estimated numerical values for all other parameters (p^*_{EU}, p^*_{LA}, r_{EU}, r_{LA}, etc.), we can determine which value of r_d yields the target correlation of ≈ 0.61 between the two network activity signals. We find that the value of r_d that achieves this is affected somewhat by the structure of the network. Table 1, Column #6 shows the values of r_d that we obtain for a range of values of $\langle k \rangle$ (average degree) and $L = 30$ interdependent links. Thus our estimate for r_d is in the range 0.10–0.16, and it is higher for smaller $\langle k \rangle$. A possible explanation is that for small values of $\langle k \rangle$, nodes have fewer neighbors, intensifying the fluctuations in the rate of external failures (we confirmed this in simulations), which increases the noise of $z(t)$ for each network. This noise component intrinsic to each network lowers the correlation between the two network signals, and a higher value of r_d is needed to compensate. Finally, by varying the total number of interdependent links L between 30 and 70, we find that the optimal r_d is only slightly affected by the value of L (as long as every node has at least one interdependent link): high values of L lower the estimate for r_d by approximately 0.01. If we relax the constraint that each node has at least one interdependent link and allow for nodes without interdependent links, this increases the estimate for r_d; some nodes are not engaged in the interaction with the other network, which weakens the interaction between the two networks, and a higher r_d is needed to compensate. In this case an *effective* r_d (a product of r_d and the fraction of nodes having at least one interdependent link) is approximately invariant with respect to L.

Once we have estimated r_d, we can correct our initial estimates for r_{EU} and r_{LA} in the estimation method #3 above, where we had initially neglected the contribution from interdependent failures. Simulating the dynamics with a nonzero r_d also gives a correction to our initial estimates for $m_{\mathrm{frac,EU}}$ and $m_{\mathrm{frac,LA}}$. Corrections for all parameters are quite small, and the final values of r_{EU}, r_{LA}, $m_{\mathrm{frac,EU}}$ and $m_{\mathrm{frac,LA}}$ are shown in Table 1, which also shows that the estimates slightly depend on $\langle k \rangle$. Supplementary Figure 1c presents an example of an actual measurement of the correlation between the two artificial network signals as a function of r_d, using numerical values from Table 1 for $\langle k \rangle = 5$ and $L = 30$. The dashed orange line indicates the correlation target value of 0.61.

The estimate of r_d and the corrections to other parameters complete our estimation for the model parameters using the real CDS records. Supplementary Figure 2a shows a typical outcome of the artificial network simulation using numerical values from Table 1, for the $\langle k \rangle = 5$ row. Supplementary Figures 2b-c show the frequency distribution histograms of the signals simulated with the $\langle k \rangle = 5$ row data, but with much longer simulation time for better statistics.

Similarity in fluctuation size structure

Our dynamical network model predicts that the typical fluctuation size of $z(t)$ is not uniform for all values of z. Supplementary Figures 2d and 2e show the average squared fluctuation $\langle [z(t) - z(t-1)]^2 \rangle$ of the activity signal as a function of z, for artificial networks A and B ($N_A = 17$, $N_B = 8$, for the parameters from Table 1, row $\langle k \rangle = 5$). The average fluctuation size shows a spike around $z \approx \frac{1}{2}$. This is a reminiscent of a critical phenomenon, since $z \approx \frac{1}{2}$ is the critical value of z below which the system is attracted to the lower single–network state, and above which it is attracted to the higher state. This behavior is best visualized by imagining a double well, where the single-network states (z_{low} and z_{high}) correspond to two wells, separated by a barrier where the top of the barrier corresponds to $z \approx \frac{1}{2}$. This is especially clear in simulations with large networks ($N > 10000$) where, depending on the initial condition, the system relaxes to either the higher state (if the initial z is above the critical value), or to the lower state (if the initial z is below the critical value). We analyze the two real CDS networks (EU and LA) and find that they also show a strong spike in average fluctuation size as a function of z (Supp. Fig. 2f-g), with the maximum at approximately the same position as in their artificial network counterparts ($z \approx \frac{1}{2}$).
